# Mg-ZIF nanozyme regulates the switch between osteogenic and lipogenic differentiation in BMSCs *via* lipid metabolism

**DOI:** 10.1186/s12944-024-02083-3

**Published:** 2024-03-25

**Authors:** Jinying Li, Yongshao Chen, Dingsheng Zha, Chunhui Wu, Xiaofen Li, Li Yang, Hui Cao, Shexing Cai, Yuebo Cai

**Affiliations:** 1 Department of Endocrinology, The Affiliated Shunde Hospital of Jinan University, Foshan, Guangdong 528300 P. R. China; 2 Department of Orthopedics Surgery, The Affiliated Shunde Hospital of Jinan University, Foshan, Guangdong 528300 P. R. China

**Keywords:** Mg-ZIF nanozyme, Osteogenic differentiation, Lipid metabolism, ROS, BMSCs, Osteoporosis

## Abstract

The accumulation of reactive oxygen species (ROS) within the bone marrow microenvironment leads to diminished osteogenic differentiation and heightened lipogenic differentiation of mesenchymal stem cells residing in the bone marrow, ultimately playing a role in the development of osteoporosis (OP). Mitigating ROS levels is a promising approach to counteracting OP. In this study, a nanozyme composed of magnesium-based zeolitic imidazolate frameworks (Mg-ZIF) was engineered to effectively scavenge ROS and alleviate OP. The results of this study indicate that Mg-ZIF exhibits significant potential in scavenging ROS and effectively promoting osteogenic differentiation of bone mesenchymal stem cells (BMSCs). Additionally, Mg-ZIF was found to inhibit the differentiation of BMSCs into adipose cells. In vivo experiments further confirmed the ability of Mg-ZIF to mitigate OP by reducing ROS levels. Mechanistically, Mg-ZIF enhances the differentiation of BMSCs into osteoblasts by upregulating lipid metabolic pathways through ROS scavenging. The results indicate that Mg-ZIF has potential as an effective therapeutic approach for the treatment of osteoporosis.

**Supplementary Information:**

The online version contains supplementary material available at 10.1186/s12944-024-02083-3.

## Introduction

Osteoporosis (OP), a prevalent degenerative bone disorder, affects an estimated 50% of women and approximately 33% of men, typically manifesting in individuals in their fifth and sixth decades of life [1–3]. OP is characterized by dysregulated bone resorption, leading to decreased bone mineral density, compromised microarchitecture, and structural deterioration, thereby increasing the susceptibility to fractures [4]. Additionally, secondary osteoporotic fractures are a significant contributor to morbidity and mortality among the elderly population [5]. The presence of these fragile fractures is associated with a notable increase in mortality rates and a substantial decline in the overall well-being of those affected [6]. Recent studies have identified a discrepancy in the differentiation of bone mesenchymal stem cells (BMSCs) in individuals with OP, wherein BMSCs within the bone marrow exhibit a preference for adipocyte differentiation over osteocyte differentiation [7, 8]. This abnormal shift towards adipogenesis and reduction in osteoblast numbers within the bone marrow contribute to the development of bone loss [9].

Reactive oxygen species (ROS) within the bone microenvironment are acknowledged as significant regulators and potential targets for the modulation of bone metabolism [10, 11]. The abnormal accumulation of ROS within the inflammatory microenvironment, along with the infiltration of inflammatory factors, plays pivotal roles in bone metabolic processes [12, 13]. Ultimately, cellular dysfunction leads to a decrease in lipid metabolism levels and stimulates the overproduction of ROS [14]. This excessive ROS impedes osteogenic differentiation while promoting lipogenic differentiation in BMSC [15]. Furthermore, the heightened lipogenic differentiation serves to enhance the production of additional ROS [16]. Recent advancements in the field of nanomedicine have introduced novel approaches for addressing OP [17]. Nanozymes, a class of nanomaterials, demonstrate catalytic properties akin to those of endogenous enzymes, providing advantages including cost-effectiveness, ease of large-scale production, exceptional stability, and prolonged shelf-life [18]. The utilization of nanozymes with characteristics akin to superoxide dismutase (SOD) and catalase (CAT) is expected to contribute to the management of OP by eradicating ROS [19]. This process is projected to improve lipid metabolism and promote the differentiation of BMSCs towards osteogenesis [20].

Zeolitic imidazolate frameworks (ZIFs), which are topological isomers synthesized using zinc ions and imidazole linkers [21, 22], are classified within the subset of metal–organic frameworks (MOFs) [23, 24]. In vitro studies have shown that ZIF exhibits stability at a pH of 7.4, a level considered physiological, for a period exceeding 15 d. Moreover, it has been established to possess biocompatibility [25]. Additionally, nanoparticles derived from ZIF demonstrate favorable cellular uptake properties [26, 27]. Consequently, the creation of a ZIF with SOD-like enzyme activity could potentially sustain its ability to scavenge reactive oxygen species over an extended period. Magnesium (Mg) deficiency has been associated with a variety of diseases, such as OP, abnormal blood lipid levels, hypertension, atherosclerosis, arrhythmias, and myocardial infarction. The hypothesis posits that reduced Mg levels in the body play a role in the development of oxidative stress [28]. Initially, hypomagnesemia leads to elevated levels of pro-inflammatory cytokines and neutrophils, as well as macrophage activation. NADPH oxidase activity results in these cells functioning as a source of superoxide anion. Additionally, it has been observed that reduced Mg levels can result in mitochondrial dysfunction and ROS [29]. Moreover, it is theorized that Mg plays a protective role in shielding cell membranes from oxidative damage [30].

**Scheme 1:**
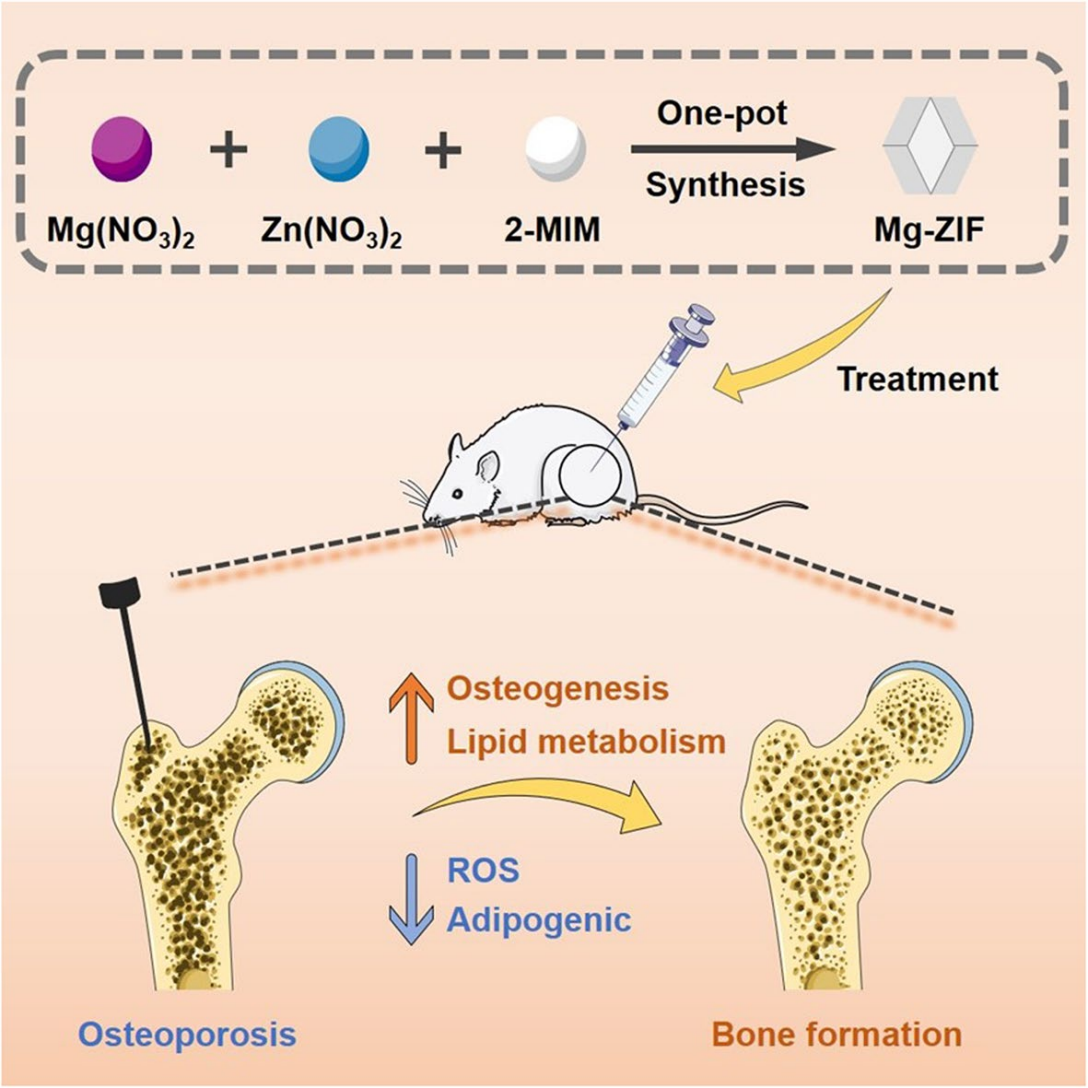
Mg-ZIF regulates osteogenic and adipogenic through lipid metabolism

In light of these findings, the proposal involves the development of a nanozyme utilizing Mg-ZIF to maintain a sustained capacity for ROS scavenging. This strategy capitalizes on the stability of ZIFs and the documented antioxidative properties of Mg, which can further fortify cells against oxidative stress-induced damage. Ultimately, this approach holds promise as a more effective therapeutic intervention for OP Scheme 1.

### Experimental section

#### Materials

All commercial reagents and solvents were of A. R. grade and were used without further purification. Mg (NO_3_)_2_·6H_2_O, Zn (NO_3_)_2_·6H_2_O, and N, N-dimethylformamide (DMF) purchased from Aladdin (Shanghai, China). CCK-8 purchased from Abbkine (Wuhan, China). Calcein AM/PI staining, DCFH-DA kit, Alkaline Phosphatase Assay Kit were purchased from Beyotime (Beijing, China). Alizarin Red S Solution obtained from Solarbio (Beijing, China). The Real-time PCR Mix and qPCR master mix were purchased from Vazyme (Nanjing, China). SOD enzyme activity was measured using commercially available kits (Beyotime, Beijing) and CAT enzyme activity was measured using a dissolved oxygen meter.

### Characterization

The single-crystal x-ray diffraction method (D8 Advance, Bruker-AXS, Germany) was employed to analyze the crystal structure, while FTIR spectroscopy (670-IR + 610-IR, Varian, USA) was used for the characterization of the chemical composition and structure. Transmission electron microscopy (Tecnai 12, Philips, Netherlands) was used to acquire information on the morphology and composition of the samples. Elemental mapping and contents were analyzed by high power transmission electron microscopy (HT7800, Hitachi, Japan).

### Cell culture

BMSCs were obtained from four male C57BL/6 J mice sourced from Jinan University's Experimental Animal Center. Briefly, the mice were killed and sterilized by immersion in 75% alcohol. Next, femurs were acquired by dissecting both lower extremities and the bone marrow was washed with PBS. Following lysis using erythrocyte lysate, the primary BMSCs were fragmented into individual cell suspensions, then cultivate for 7 d and use for subsequent cell experiments after reaching full growth. The BMSCs were identified by LepR immunostaining (Fig. S1).

### Synthesis of Mg-ZIF

A typical one-pot method was used to synthesize ZIF and Mg-ZIF [31]. In brief, after dissolving Mg (NO_3_)_2_ · 6H_2_O (1.0 mmol, 0.25641 g) and Zn (NO_3_)_2_ · 6H_2_O (1.0 mmol, 0.29749) in DMF, the mixture is stirred for a duration of 20 min. Subsequently, 10 mL of a N, N-Dimethylformamide (DMF) solution containing dimethylimidazole (6.0 mmol, 0.4926) was incrementally added to the reaction mixture. The resulting mixture was subjected to overnight stirring, followed by centrifugation at 10,000 rpm for 10 min to isolate the precipitate. The precipitate was then subjected to three washes with alcohol and subsequently dried for 12 h in a drying oven set at 60 ℃. With respect to the synthesis of ZIF, Mg (NO_3_)_2_ · 6H_2_O is replaced by 1 mmol of Zn (NO_3_)_2_ · 6H_2_O in the first step, and the rest of the steps remain the same.

### Cell proliferation and live/dead staining assay

BMSCs were cultured on 96-well plates at a cell density of 8000 cells per well. The cells were then cultured for 24 h to facilitate cell adhesion. The viability of cells was assessed by employing the Cell Counting Kit 8 (Sigma-Aldrich, Shanghai, China) as per the instructions provided by the manufacturer, following co-incubation with nanoparticles for 1, 3, and 7 d. Likewise, the viability of cells was assessed by employing the live/dead staining kit in accordance with the guidelines provided by the manufacturer. Calcein AM/PI double staining (Beyotime) serves as a cell death assay to quantify the population of live and dead cells. In short, cells were subjected to testing with Calcein AM (for viable cells) and PI (for non-viable cells) solutions at 37 °C for a duration of 30 min in the absence of light. Subsequently, the resulting image was analyzed using a fluorescence microscope.

### Assessment of osteogenic activity of BMSCs

Regarding the ALP activity assay (Beyotime, Beijing), BMSCs were cultured with osteogenic differentiation medium for 7 d according to control, H_2_O_2_ (200 μM), H_2_O_2_ + ZIF, and H_2_O_2_ + Mg-ZIF grouping, and ALP activity was assayed using a kit. Next, to confirm mineralization, BMSCs were induced for 21 d, and these cells were fixed using 70% ethanol and then stained for mineralized nodule formation using 2% alizarin red.

### Induction of adipogenesis of BMSCs

To stimulate adipogenesis, BMSCs were seeded into plates with a density of 4 × 10^4^ per well. Adipogenesis induction was performed when the cell density reached about 90% according to different groupings. The medium for inducing adipogenesis was split into two solutions, adipogenesis A and B. Adipogenic solution A consisted of a full medium with the addition of 0.5 M dexamethasone, 0.25 mM IBMX, 0.2 mM rosiglitazone, and 10 μg/mL insulin. The solution B for adipogenesis consisted of a fully supplemented medium containing 10 μg/mL of insulin. Solution A was utilized for a duration of 3 d during the initiation of adipogenesis, followed by the utilization of solution B for 1 d. After 21 d of inducing adipogenesis, staining for adipogenesis was conducted.

### Analysis using quantitative real-time polymerase chain reaction (qRT-PCR)

First, BMSCs were inoculated at 10^5^ per well in 6-well plates and cultured with different treatment groups for 3 d after 24 h of adherence. Subsequently, total RNA was isolated from the cells through the addition of TRIzol reagent (Beyotime, China), followed by the generation of cDNA using TaKaRa reverse transcription reagent (TaKaRa, Shiga, Japan). Subsequently, a qPCR assay was conducted to analyze the expression of pertinent genes. Real-time PCR was performed using a 20-μL reaction mixture containing SYBR Green qPCR Master Mix, forward and reverse primers, DEPC water, and cDNA template. Femoral head tissue was mechanically disrupted and ground with liquid nitrogen for RNA extraction, followed by cDNA synthesis. Real-time PCR was conducted using a Light Cycler instrument (Roche, Basel, Switzerland). The qPCR conditions were as follows: 1 cycle of initial denaturation at 95 °C for 4 min followed by 40 cycles of denaturation at 95 °C for 15 s, annealing at 60 °C for 30 s, and elongation at 72 °C for 30 s. The 2^−ΔΔCt^ method was applied and gene expression was normalized with Actin. Primers are listed in Table S1 [32–39].

### Animal model

Female C57BL/6 J mice, aged 5 weeks, were acclimatized and fed for 1 week before being divided into groups for ovariectomy (OVX) or sham surgery. Following OVX procedure, a single injection of nanozyme was administered into the bone marrow cavity immediately at a dosage of 5 mg/kg, and no further injections were given during the subsequent period. The groups included sham, OVX, OVX + ZIF, and OVX + Mg-ZIF. The femur was taken 6 weeks after treatment for follow-up experiment. Over a period of 6 weeks, animals were housed in cages containing 5 individuals each and provided with food and shelter under standardized conditions, including a temperature of 21 °C and a relative humidity of 55%, as well as a 12 h light/dark cycle. All animal procedures were conducted in accordance with the guidelines set forth by the Institutional Animal Care and Use Committee of Jinan University (Approval number: IACUC-20230617–15) and adhered to the "Guide for the Care and Use of Laboratory Animals" established by the National Institute of Health in China.

### Micro-computed tomography reconstructions, immunofluorescence and Trap staining

Following the euthanization of the mice, the femurs were gathered and immersed in 10% neutral buffered formalin for a duration of 24 h. Place the femur vertically in the tube sample holder, and then fix it in the foam to avoid slight movement that may affect the high-resolution scanning. By utilizing the SkyScan1176 micro-CT, one can examine the alterations in the microstructure of the femur and acquire three-dimensional reconstructed visuals. Bone parameters were then analyzed by CTAn on the 3D reconstructed images of the bone. Femurs were decalcified in 10% EDTA for 10 d, embedded, cut into 4 µm sections, and stained for OCN and Trap.

### Statistical analysis

Experiments were repeated three times and data were presented as mean ± SD. Statistical analyses were conducted using GraphPad Prism 9 software with significance set at *p* < 0.05. (* *P* < 0.05, ** *P* < 0.01, *** *P* < 0.001).

## Result and discussion

### Synthesis and characterization of Mg-ZIF

**Fig. 1:**
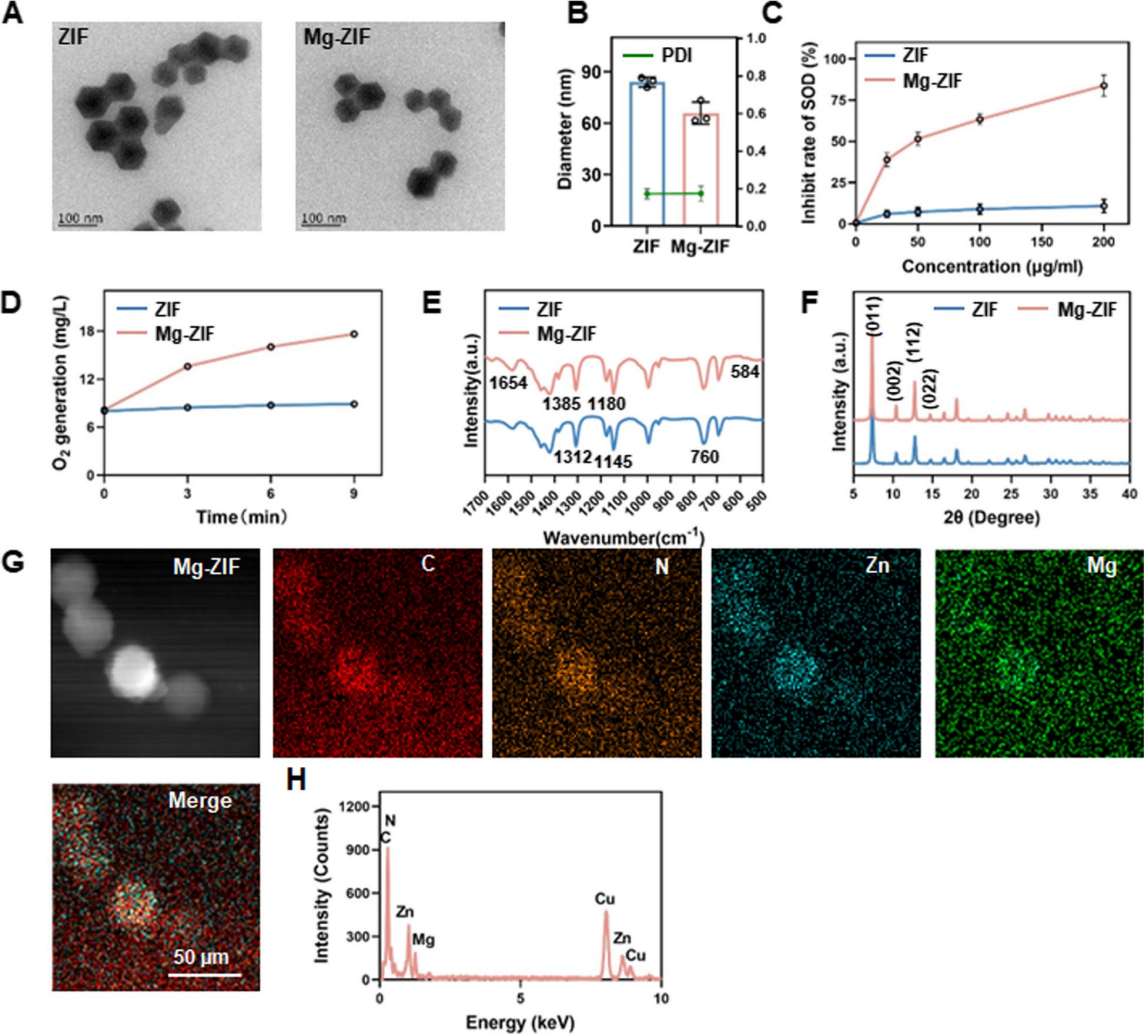
Synthesis and characteristics of Mg-ZIF. **A** TEM images of ZIF and Mg-ZIF. **B** Size and distribution of nanoparticles produced. Enzyme-like activities of ZIF and Mg-ZIF nanoparticles: SOD enzyme activity (**C**) and CAT enzyme activity (**D**). **E** FTIR and XRD (**F**) patterns of ZIF (blue) and Mg-ZIF (pink). **G**, **H** Elemental mapping and EDS confirm the presence of C, N, Mg, and Zn in Mg-ZIF

MOFs are composed of metal ions or clusters and organic linkers. ZIF, a subset of zeolite imidazolate frameworks (ZIFs), consists of zinc ions and 2-methylimidazole, exhibiting favorable stability, biocompatibility, and cellular uptake [40, 41]. This study aimed to explore the potential for synthesizing ZIFs with enzyme-like activity by varying the metal ion composition through a one-pot method. Transmission electron microscopy (TEM) images revealed well-defined morphologies for both ZIF and Mg-ZIF, consistent with previous descriptions (Fig. 1A) [42]. The Malvern particle size analysis revealed that the average hydrodynamic diameters of ZIF and Mg-ZIF were 83.97 ± 2.91 nm and 65.86 ± 6.44 nm, respectively. Additionally, both nanoparticles exhibited a low particle dispersion index (PDI), suggesting effective dispersion (Fig. 1B).

In this study, the SOD and CAT-like enzyme activities of magnesium-based zeolitic imidazolate framework (Mg-ZIF) was investigated. The SOD activity of Mg-ZIF nanozymes was assessed using a SOD assay kit, revealing an increase in SOD enzyme activity with higher nanozyme concentrations. At a concentration of 100 μg/mL, the nanozyme demonstrated an inhibition rate of approximately 85% (Fig. 1C). The primary role of the SOD enzyme is to facilitate the conversion of superoxide anion radicals into hydrogen peroxide and oxygen, both of which can be detrimental to cellular health. CAT exhibits efficient decomposition of H_2_O_2_ into H_2_O and O_2_ [43]. In this study, O_2_ production was measured in the presence and absence of Mg-ZIF using a dissolved oxygen meter, revealing that the synthesized Mg-ZIF displayed significant CAT-like enzyme activity (Fig. 1D). These findings indicate that the synthesized nanozymes possess notable SOD and CAT-like enzyme activities, suggesting their potential for mitigating excessive ROS levels at inflammatory sites.

In order to corroborate the crystal morphology, ZIF and Mg-ZIF were analyzed using Fourier transform infrared spectroscopy (FTIR), revealing consistent characteristic peaks (Fig. 1E). The observation found various vibrations on the nanoparticles, including N–H wagging, ring expansion, and methyl bending at specific frequencies. Additionally, C-N stretching, N–H swinging, C = N out of plane bending, and N–H bending were also detected. The findings presented in Fig. 1F demonstrate that powder X-ray diffraction (XRD) analysis revealed a similarity between the data obtained for Mg-ZIF and conventionally prepared ZIF8. This suggests that the incorporation of Mg does not alter the crystal morphology of ZIF. Additionally, the presence of Zn and Mg elements in the samples was confirmed through the use of EDS and element mapping, as depicted in Fig. 1G and H, respectively.

Mg-ZIF nanozymes possess good biocompatibility and ROS scavenging ability.

**Fig. 2:**
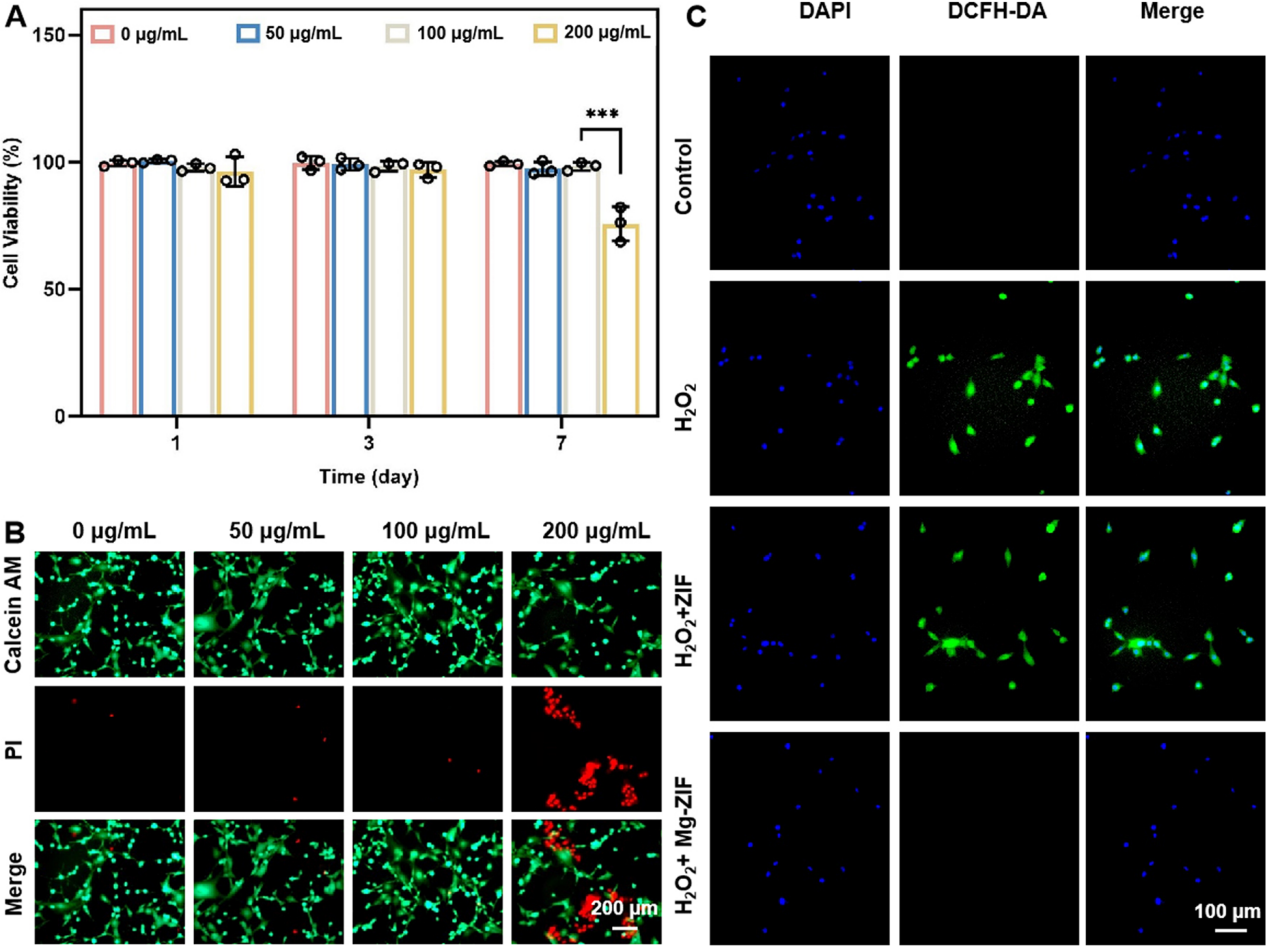
Biocompatibility and anti-H_2_O_2_ effect of Mg-ZIF. **A**, **B** Cytotoxicity of Mg-ZIF against BMSCs. **C** Mg-ZIF alleviated ROS accumulation caused by H_2_O_2_. Data presented as mean ± SD (*n* = 3), ***P < 0.001

Prior to assessing the enzymatic properties of nanozymes, an investigation into the biocompatibility of Mg-ZIF with BMSCs was conducted. Utilizing the CCK-8 assay, it was determined that Mg-ZIF exhibited favorable cell viability within the concentration range of 0 to 100 μg/mL (Fig. 2A). Additionally, cell live-dead staining demonstrated no significant cell mortality after co-incubation of BMSCs with Mg-ZIF at concentrations ranging from 0 to 100 μg/mL for a duration of 7 d (Fig. 2B). These findings suggest that Mg-ZIF displays commendable biocompatibility. The ROS scavenging capability of Mg-ZIF nanozymes was subsequently demonstrated through a co-culture experiment involving BMSCs treated with 100 μg/mL Mg-ZIF and 200 μM H_2_O_2_ for a duration of 24 h. The administration of this treatment led to a notable decrease in cellular ROS levels in comparison to the control groups (Fig. 2C), suggesting that Mg-ZIF nanozymes possess favorable biocompatibility and ROS scavenging properties.

### Mg-ZIF promotes osteogenic differentiation and suppresses lipogenic differentiation of BMSCs by upregulating lipid metabolism

**Fig. 3:**
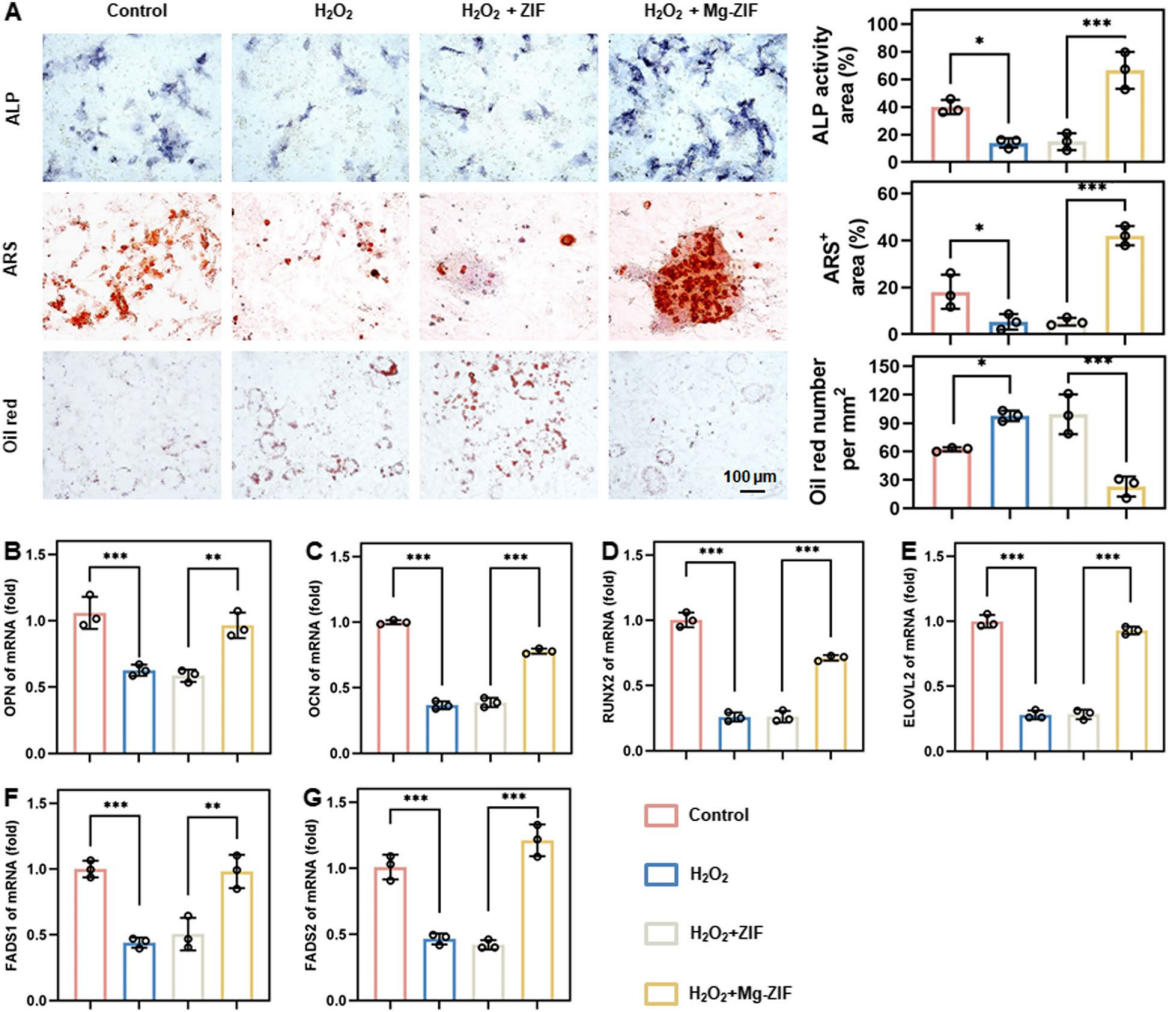
Mg-ZIF nanozymes alter the differentiation fate of BMSCs by upregulating lipid metabolism. **A** Images and analysis of ALP, ARS, and Oil red staining in various treatment groups. **B**–**D** Levels of osteogenic differentiation (OPN, OCN, and RUNX2) gene expression in BMSCs. **E**–**G** Levels of lipid metabolism (ELOVL2, FADS1, and FADS2) gene expression in BMSCs. Data presented as mean ± SD; *n* = 3, *P < 0.05, **P < 0.01, *** *P* < 0.001

The regulatory effect of Mg-ZIF on BMSCs was analyzed by co-incubating with osteogenic induction medium. It was observed that BMSCs treated with Mg-ZIF exhibited elevated alkaline phosphatase (ALP) activity compared to other groups (Fig. 3A). Additionally, the presence of Mg-ZIF resulted in the formation of polymineralized nodules on 21 d, indicating its potential to enhance osteogenic differentiation of BMSCs. Additionally, qRT-PCR analysis showed that Mg-ZIF greatly enhanced BMSCs' osteogenic differentiation compared to the H_2_O_2_ group (Fig. 3B–D). To assess the influence of Mg-ZIF on adipogenesis, BMSCs were cultured in an adipogenic-inducing medium supplemented with diverse substances. The findings revealed that the inclusion of Mg-ZIF impeded the formation of lipid droplets in BMSCs, as depicted in Fig. 3A, suggesting the efficacy of Mg-ZIF in suppressing the adipogenic differentiation of BMSCs. Furthermore, it has been demonstrated that disruption of lipid metabolism can result in aberrant accumulation of ROS. In order to elucidate the regulatory mechanism of Mg-ZIF in BMSCs, an analysis of lipid metabolism levels within the cells was conducted. qRT-PCR data showed that H_2_O_2_ significantly reduced the expression of important lipid metabolism genes, including ELOVL2, FADS1, and FADS2 (Fig. 3E–G). Importantly, the introduction of Mg-ZIF resulted in a significant upregulation of these aforementioned genes. These results indicate that Mg-ZIF boosts BMSCs' bone-forming ability by reducing ROS levels through lipid metabolism activation.

### Mg-ZIF alleviates osteoporosis in ovariectomy (OVX) mice by upregulating lipid metabolism

**Fig. 4:**
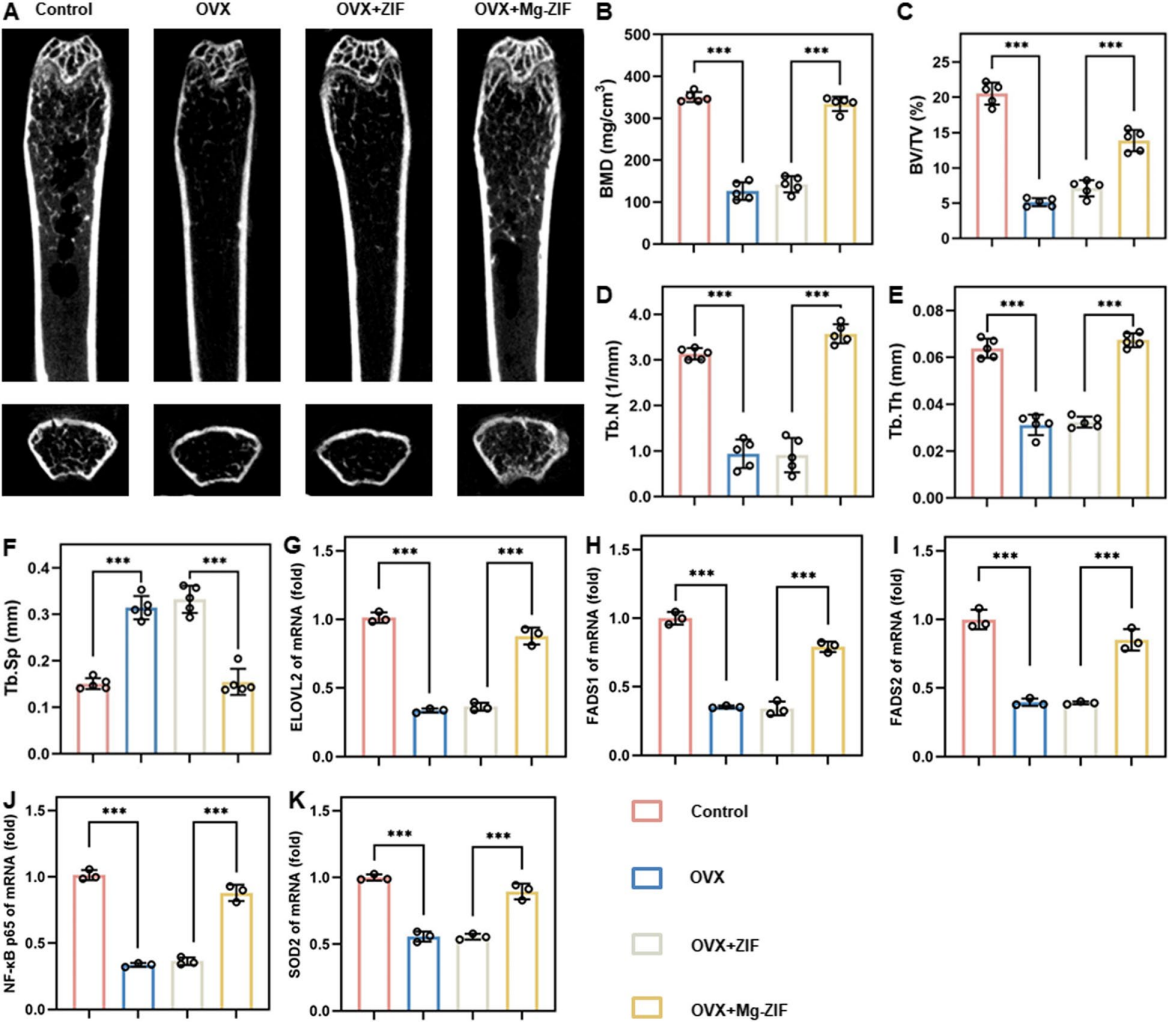
Mg-ZIF nanozymes alleviate OP. **A** Representative micro-CT images. **B**–**F** A quantitative analysis was conducted on bone mineral density (BMD), bone trabecular volume (BV/TV), number of trabeculae (Tb.N), thickness of trabeculae (Tb.Th), and separation of trabeculae (Tb.Sp) with a sample size of *n* = 5. **G**–**K** Expression of lipid metabolism-related and oxidative stress-related genes in BMSCs. Data presented as mean ± SD; *n* = 3, **P < 0.01, ***P < 0.001

**Fig. 5:**
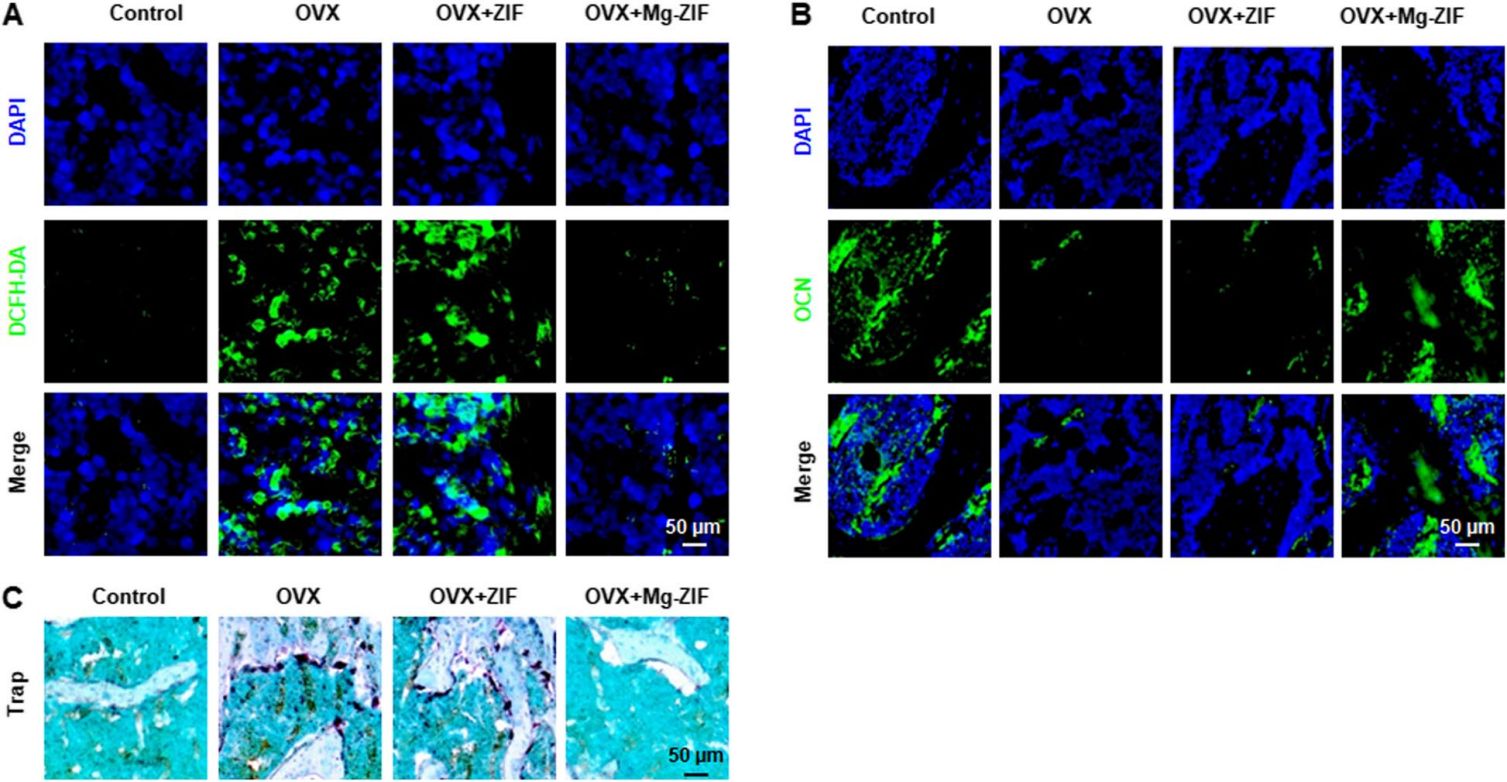
Mg-ZIF nanozymes efficiently remove ROS, support bone growth, and prevent bone breakdown. **A**, **B** The DCFH-DA and immunofluorescence staining images of mouse femurs after different treatments. **C** The Trap staining images of mouse femurs after different treatments

The effectiveness of Mg-ZIF nanoenzymes in alleviating osteoporosis (OP) was evaluated by administering them into the femoral bone marrow cavity of OVX mice. Analysis using micro-computed tomography (micro-CT) and three-dimensional reconstruction revealed a notable reduction in bone volume in OVX and OVX + ZIF mice compared to those in the Mg-ZIF group, as indicated by decreases in bone mineral density (BMD), bone volume fraction (BV/TV), number of trabeculae (Tb. N), and trabecular thickness (Tb. Th) (Fig. 4A–F). The decrease in trabecular spacing (Tb. Sp) noted in Mg-ZIF indicates a significant anti-osteoporotic impact, as illustrated in Fig. 4B–F. BMSCs were employed to evaluate the transcription of genes associated with lipid metabolism and oxidative stress. Quantitative polymerase chain reaction (qPCR) analysis confirmed the in vitro findings, showing a significant decrease in ELOVL2, FADS1, and FADS2 expression in OVX mice bone marrow, while their expression was notably increased in Mg-ZIF treated mice (Fig. 4F–H). Concurrently, the role of superoxide dismutase 2 (SOD2) genes in cellular defense against oxidative stress and maintenance of cellular health has been established. Additionally, NFκB genes are closely linked to the upregulation of oxidative stress. Therefore, the study analyzed the levels of SOD2 and NFκB genes in mouse femur sections. Notably, the expression of NFκB gene exhibited a notable decrease in the Mg-ZIF group, while the expression of ARE was significantly elevated. These findings indicate that Mg-ZIF demonstrates enhanced antioxidant stress capabilities in an in vivo setting. Furthermore, the DCFH-DA probe is commonly utilized for evaluating oxidative stress levels. Therefore, an assessment of the antioxidant activity of Mg-ZIF nanoenzymes in OVX mice using the DCFH-DA probe was conducted. The findings indicated a notable decrease in DCFH-DA levels in mice treated with Mg-ZIF compared to those in other treatment groups, suggesting a significant reduction in oxidative stress levels (Fig. 5A). Moreover, the findings from immunofluorescence staining and Trap staining suggest that mice administered with Mg-ZIF demonstrated elevated OCN expression and reduced osteoclast presence compared to other treatment cohorts. This observation is consistent with the hypothesis that Mg-ZIF nanozymes promote osteogenic differentiation of BMSCs by reducing ROS levels, as depicted in Fig. 5B, C. These findings suggest that Mg-ZIF nanozymes hold promise as a viable approach for mitigating OP.

### Study strengths and limitations

This work includes the following advantages: firstly, the synthesized Mg-ZIF nanozyme had good ROS scavenging ability, which can effectively clear ROS in vitro and in vivo. Then, Mg-ZIF upregulated lipid metabolism by inhibiting ROS, thereby inhibiting the adipogenic differentiation of BMSCs and promoting their osteogenesis. Finally, Mg-ZIF nanozymes exhibited excellent anti osteoporosis efficacy in vivo. However, this study still had certain limitations, such as unclear molecular mechanisms by which nanozymes regulate lipid metabolism. It is necessary to further explore the molecular mechanisms by which nanozymes regulate lipid metabolism in future research.

## Conclusions

In summary, nanoparticles with enzyme-like activity were synthesized for anti-osteoporosis therapy through the incorporation of Mg-ZIF. The resulting Mg-ZIF exhibited robust ROS scavenging capabilities and promoted osteogenic differentiation of BMSCs while inhibiting their adipogenic differentiation. Mechanistic investigations revealed that Mg-ZIF attenuated ROS generation by upregulating lipid metabolic pathways, thereby effectively mitigating the progression of OP. While traditional anti bone resorption drugs such as bisphosphonates and denosumab, as well as synthetic metabolic drugs, have been utilized in clinical settings to mitigate bone loss, they have shown limited consideration for the impact of lipid metabolism in OP. Consequently, the incorporation of Mg-ZIF alongside these conventional anti bone resorption drugs may yield a more efficacious anti-OP cascade effect.

### Supplementary Information

**Supplementary Material 1.**

## Data Availability

Data is provided within the manuscript or supplementary information files.
