# Supplementary material for: Mg-ZIF nanozyme regulates the switch between osteogenic and lipogenic differentiation in BMSCs via lipid metabolism
Source: Lipids Health Dis. 2024 Mar 25;23:88. doi: 10.1186/s12944-024-02083-3 (PMC10962101; doi:10.1186/s12944-024-02083-3)
Supplement: Supplementary file 1 — Supplementary Material 1. [file 12944_2024_2083_MOESM1_ESM.docx]

**Supplementary information**

**Mg-ZIF nanozyme regulates the switch between osteogenic and lipogenic differentiation in BMSCs *via* lipid metabolism**

Jinying Li ^1#^, Yongshao Chen ^2#^, Dingsheng Zha ^2^, Chunhui Wu ^2^, Xiaofen Li ^2^, Li Yang ^2^, Hui Cao ^2^, Shexing Cai ^2^ and Yuebo Cai ^2^*

^1^ Department of Endocrinology, the Affiliated Shunde Hospital of Jinan University, Foshan, Guangdong, 528300, P. R.China.

^2^ Department of Orthopedics Surgery, the Affiliated Shunde Hospital of Jinan University, Foshan, Guangdong, 528300, P. R.China.

* Corresponding author.

E-mail address: bocai315@163.com (Y. Cai).

^#^ These authors have contributed equally to this work


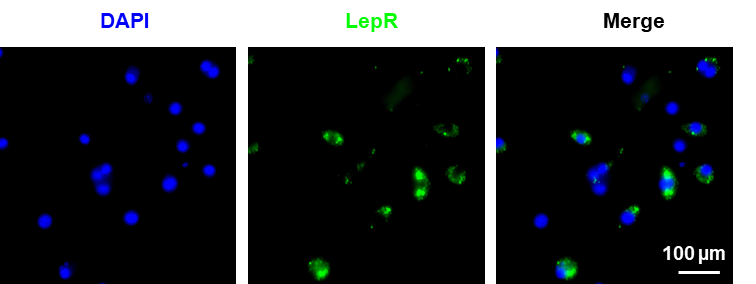


Fig. S1. the characterization of BMSCs.

Table S1. Primers for qPCR analysis (species, mouse)

| Genes | Sequences (5’-3’) | | GenBank  accession no. | Annealing temperature |
| --- | --- | --- | --- | --- |
| OPN | F:GCAGTCTTCTGCGGCAGGCA | R:GGGTCAGGCACCAGCCATGTG | NM_001204203.1 | 60 °C |
| OCN | F:GCAATAAGGTAGTGAACAGACTCC | R:GTTTGTAGGCGGTCTTCAAGC | NM_001032298.3 | 60 °C |
| RUNX2 | F:CTCTTCTGGAGCCGTTTATGT | R:GTTTCTTAGGGTCTTGGAGTGA | NM_001271630.2 | 60 °C |
| ELOVL2 | F:TCACGTACCTGCTCTCGATATGGC | R:GATGAGCTCCACCAGCATATACGC | NM_001311121.1 | 60 °C |
| FADS1 | F:CCAGCTTTGAACCCACCAA | R:CATGAGGCCCATTCGCTCTA | NM_146094.2 | 60 °C |
| FADS2 | F:CTCTCGTACTTCGGCACTGG | R:GCCATAGTCATGTTGCAGCC | NM_019699.2 | 60 °C |
| NF-κB p65 | F:AAGCACAGATACCACCAAGACAC | R:CGCACTGCATTCAAGTCATAGTC | NM_001402548.1 | 60 °C |
| SOD2 | F:CCAAGGGAGATGTTACAACTCAG | R:GGGCTCAGGTTTGTCCAGAA | NM_013671.3 | 60 °C |
| ACTIN | F:GGCTGTATTCCCCTCCATCG | R:CCAGTTGGTAACAATGCCATGT | NM_007393.5 | 60 °C |
